# Rapid adaptation accelerates competitive suppression in a parasite community

**DOI:** 10.1093/ismejo/wrag114

**Published:** 2026-06-13

**Authors:** Samuel T E Greenrod, Daniel Cazares, Weronika Ślesak, Tobias E Hector, R Craig MacLean, Kayla C King

**Affiliations:** Department of Biology, University of Oxford, Oxford, OX1 3EL, United Kingdom; Department of Biology, University of Oxford, Oxford, OX1 3EL, United Kingdom; Department of Biology, University of Oxford, Oxford, OX1 3EL, United Kingdom; Department of Biology, University of Oxford, Oxford, OX1 3EL, United Kingdom; Department of Biology, University of Oxford, Oxford, OX1 3EL, United Kingdom; All Souls College, High Street, Oxford, OX1 4AL, United Kingdom; Department of Biology, University of Oxford, Oxford, OX1 3EL, United Kingdom; Department of Zoology, University of British Columbia, Vancouver, V6T 1Z4, Canada; Department of Microbiology and Immunology, University of British Columbia, Vancouver, V6T 1Z3, Canada

**Keywords:** phage, bacteria, temperature, evolution, competition, community, parasite, adaptation

## Abstract

Environmental stress leads to changes in community composition by altering competitive hierarchies and pushing taxa towards extinction. Parasites and their communities are particularly vulnerable to stress due to environmental sensitivity of infection steps and dependence on host fitness. Parasite populations might avoid extinction through evolutionary rescue—whereby rapid adaptation to stress enables persistence—but the impacts of adaptation to stress on parasite communities remain unclear. Here, we study the evolutionary and ecological impact of thermal stress in a simple parasite community by propagating populations of two viral parasites (bacteriophages φ14-1 and φLUZ19) of *Pseudomonas aeruginosa* in monoculture and co-culture under two thermal conditions: a control temperature (37°C) and a high temperature that restricts φ14-1 growth (42°C). We show that rapid thermal adaptation of φ14-1 facilitated persistence in monoculture. Rescue of this phage in co-culture made it a superior competitor, and it replaced φLUZ19 as the dominant phage at high temperature. We determine that thermal adaptation occurred through mutations in genes linked to attachment to bacterial hosts and within-host replication. We also show that competitive suppression by φ14-1 constrained φLUZ19 molecular evolution. Our findings suggest that rapid adaptation to environmental stress can prevent the extinction of some parasites but may inadvertently destabilise the community and facilitate further species loss. This work underscores the need to take an eco-evolutionary approach to predict the responses of communities to global climate change.

## Introduction

Environmental stress is a primary driver of biodiversity loss [1, 2]. On an ecological timescale, only those species with environmental optima and tolerance ranges best aligned with prevailing conditions can persist [3–5]. As community composition shifts towards fewer species, the probability of extinction events [6] and ecological tipping points [7] is heightened. On an evolutionary timescale, however, biodiversity could be maintained via evolutionary rescue, whereby rapid adaptation to environmental stress facilitates population recovery [7, 8]. Evolutionary rescue has been shown to prevent diversity loss in communities and increase the prevalence of rare taxa whose relative fitness increases following adaptation [9–11]. Alternatively, evolutionary rescue can alter the competitive hierarchy in communities and drive competitor decline and exclusion [12–14]. Understanding how environmental stress affects biodiversity requires consideration of both ecological and evolutionary dynamics in communities [15].

Parasite communities are expected to be among those most disrupted by climate change, with consequences for global disease dynamics and ecosystem stability [16]. Parasite diversity may decrease with thermal stress through widening interspecific fitness differences [17, 18] and increasing host resistance [19, 20]. Diversity can also be lost through thermal alterations to parasite life-history strategies [20, 21] or a reduction in niche differences [22, 23]. Bacteriophages (hereafter referred to as phages), parasites of bacteria, offer a powerful model system to study evolutionary responses to stress in parasite communities due to their rapid evolution rates and frequent competitive interactions [24, 25]. Phages can be highly sensitive to thermal extremes (see [26] for review) and, in isolation, have been shown to avoid thermal extinction through rapid adaptation [17, 27, 28]. However, the evolutionary responses to thermal stress in phage communities are more complex. Modelling has indicated that competitive interactions in free-living microbial communities may constrain adaptation by reducing population growth rates and mutational supply [5, 29]. These findings have since been confirmed in bacterial populations through experimental studies [30, 31]. In phage communities, adaptation may instead be promoted with competition either through recombination during co-infections [32] or co-selection with thermal stress on the same phage life-history traits [26, 33]. Competitive interactions may thus accelerate phage adaptation and increase the risk of competitor exclusion in response to stressful temperatures.

**Figure 1:**
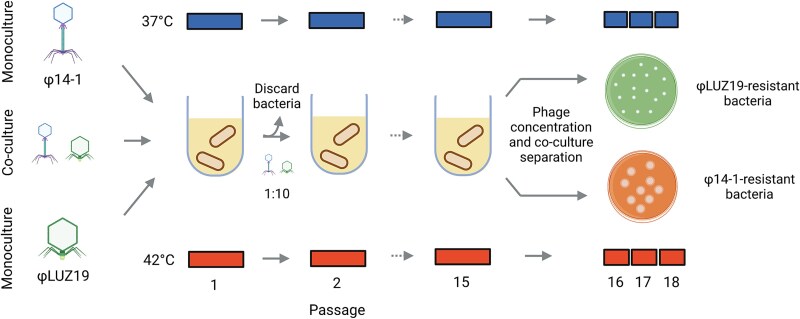
Overview of phage experimental evolution framework. Phages evolved in monoculture or co-culture at 37°C or 42°C for 15 passages. At the end of each passage, phages were isolated from lysates and used to infect fresh, evolutionarily static bacterial hosts (passages 1–15). At the end of the selection experiment, phages were concentrated and co-cultures separated through three rounds of confluent lysis plating on phage-resistant bacteria (passages 16–18) under the same thermal regime as earlier passages. Phage icons illustrate the two different phages used in the experiments (φ14-1, myovirus; φLUZ19, autographivirus) [34] and are used hereafter to refer to phages in figures. The figure was created using BioRender.

Rapid adaptation is predicted to increase the absolute fitness of phage species during warming and prevent extinction. Although competition in phage communities could constrain evolution by limiting mutational supply [29], rapid adaptation could skew the competitive hierarchy towards the rescued, thereby accelerating competitive exclusion. To test these predictions, we passaged two lytic phages (thermal generalist φLUZ19 and specialist φ14-1) through evolutionarily static populations of a bacterial host, *Pseudomonas aeruginosa* (Fig. 1). These phages are obligate killers; following phage attachment and infection of host cells, they immediately initiate replication and host cell lysis (death). Phages were evolved under a control temperature (37°C) or high temperature that restricts the growth of the φ14-1 phage (42°C) [18] in monoculture or co-culture. We tested for effects on phage growth rates and competitiveness by conducting phenotypic assays of phage infectivity in the absence and presence of a phage competitor. We also used sequencing to measure changes in the genomic composition of phage populations through time.

## Methods and materials

### Strains, storage, and culture conditions

*Pseudomonas aeruginosa* PAO1 (hereafter referred to as PAO1) was used with two lytic bacteriophages: φLUZ19 [35, 36] and φ14-1 [37]. These phages have been used to test several ecological and evolutionary hypotheses at 37°C [18, 38–41]. The phages were also selected based on their distinct responses to temperature: φLUZ19 has high growth rates at 37°C and 42°C, whereas φ14-1 has restricted growth at 42°C [18]. Bacterial stocks and phage lysates were prepared as in ref [18].

### Experimental evolution

A schematic of the experimental evolution framework is shown in Fig. 1. Each of the 15 evolutionary passages was made across three phage treatments (φLUZ19 and φ14-1 monocultures and co-culture) and two temperatures (37°C and 42°C). Each treatment consisted of six independent replicate populations started from a single ancestral lysate.

Phages were propagated without shaking with a nonevolving ancestral PAO1 bacterial host. For the initial passage, ancestral phage lysates were diluted to 10^8^ PFU/ml, and 300 μl were added to 2.7 ml 10^8^ CFU/ml bacterial culture in loose-lid 14 ml Falcon tubes. Phage co-culture populations were prepared by combining 150 μl each of φLUZ19 and φ14-1 10^8^ PFU/ml stocks prior to mixing with bacteria. Phages were added at a 1:1 ratio to mimic a phage community that is already stable and at the maximum level of biodiversity possible with a two-species system. The initial passage phage densities were ~ 10^7^ PFU/ml, resulting in a phage/bacteria ratio (multiplicity of infection, MOI) = ~0.1. Phage φ14-1 had slightly higher starting densities (3.8 × 10^7^ vs 1.1 × 10^7^ PFU/ml in φLUZ19) due to minor dilution variation. Bacterial culture densities were standardized using optical density (OD595) based on a CFU:OD standard curve (Fig. S1). Following the addition of bacterial cultures, tubes were incubated statically at 37°C or 42°C in circulating water baths for 8 h.

After each passage, phage lysates were centrifuged at 3095× *g* for 5 min to pellet remaining bacterial cells. Phage lysates were then sterile-filtered using 0.2 μm syringe filters into 2 ml cryotubes and stored at 4°C. At the beginning of each passage, 2.7 ml of fresh ancestral PAO1 was seeded with 300 μl of unattached phage from the preceding passage’s filtered phage lysate. Phage densities were not controlled after the initial passage.

### Phage quantification

Phage titres were determined via the double-layer overlay method [42] following the same protocol as in ref [18]. Briefly, bacterial lawns were prepared by mixing 10 ml of melted top agar with 300 μl of a *P. aeruginosa* PAO1 overnight culture. Phage stocks were serially diluted, and 10 μl was spotted onto bacterial lawns. After incubating plates for 6–8 h at 37°C, spots with the highest number of discernible plaques were counted. Top-agar bacterial lawns were seeded with φLUZ19-resistant and φ14-1-resistant PAO1 strains to quantify φ14-1 and φLUZ19 populations, respectively. Phage-resistant PAO1 strains were generated by spotting high-titre phage stocks (~10^10^ PFU/ml) onto wild-type PAO1 top-agar lawns. Plates were then incubated for ~48 h or until colonies started to grow on top of phage clearance zones. Five colonies were picked for each phage and were restreaked twice before being used to seed 10 ml Luria–Bertani (LB) (Lennox) and grown statically at 37°C in 50 ml Falcon tubes. The absence of phage in resistant cultures was determined through sterile filtration of the bacterial supernatant and spotting onto ancestral PAO1 bacterial lawns.

Phage resistance was confirmed through the absence of plaques when spotting high-titre phage stocks onto lawns seeded with each resistant mutant. One resistant mutant was selected for each phage by spotting phage stocks of known concentration (based on wild-type PAO1 estimates) and selecting the mutant that had the closest plaque count, turbidity, and size to the wild-type PAO1. Phage-resistant PAO1 mutants underwent whole-genome hybrid (long- and short-read) sequencing and variant calling (see DNA extraction and sequencing, and sequence analysis for methods). Although both PAO1 mutants had multiple mutations compared to wild-type (Table S3), we identified mutations that were previously linked to phage resistance. φLUZ19-resistant PAO1 had a mutation in a GspL type-II secretion system protein (BlastP: 99.5% similarity and 100% query cover); *Gsp* gene mutations have previously shown to provide resistance to type-IV pilus dependent phages [43]. φ14-1-resistant PAO1 had a mutation in a glycosyltransferase (BlastP: 100% similarity and query cover); glycosyltransferase mutations have been shown to provide resistance to LPS-dependent phages [44]. To maintain comparability, both monoculture and co-culture populations were quantified using resistant PAO1 strains.

Changes in phage counts during the evolution experiment could reflect evolutionary changes in efficiency of plaque formation rather than changes in phage densities. The efficiencies of plaque formation of the passage 15 evolved and ancestral phage populations were found to be similar when tested on the ancestral and resistant PAO1 strains separately (Fig. S2). This outcome confirmed that phage counts reflected changing phage densities.

### Phage separation and concentration

Evolved phage populations needed to be concentrated and purified to separate co-cultures and create high-titre stocks for phage population sequencing (Fig. 1). High-titre, pure phage stocks were made using a selective confluent lysis double-layer overlay method. Briefly, bacteria-phage lawns were prepared by mixing 180 μl of either φLUZ19-resistant or φ14-1-resistant PAO1 overnight cultures (~3 × 10^8^ CFU/ml) with 30 μl of phage lysate diluted to 10^8^ PFU/ml. Initial bacterial culture densities were ~ 10^8^ CFU/ml, and phage densities were ~ 10^6^ PFU/ml, MOI = ~0.01. Bacteria-phage mixtures were left at room temperature for ~10 min to allow phage adsorption after which 5 ml of molten top agar (~40°C) was added, and agar was poured onto prefilled LB-agar plates. Plates were incubated for ~20 h at temperatures appropriate for each evolved population: 37°C for 37°C evolved phage populations, 42°C for 42°C evolved populations. After incubation, top agar was scraped off plates into 15 ml Falcon tubes containing 5 ml phage buffer [NaCl (100 mM), MgSO_4_ (10 mM), CaCl_2_ (5 mM), Tris–HCl (pH 8) (50 mM), Gelatin (0.01%)]. Tubes were incubated at 4°C on a rotating carousel shaker at 10 rpm for 24 h to extract phage from top agar. Phages were separated from bacteria and agar by centrifuging tubes at 6000× *g* for 10 min followed by sterile-filtering. The purification/concentration process was repeated three times to remove nonfocal phages, and purity was assessed based on the absence of competitor plaques following high-titre spotting. To ensure comparability, monoculture populations underwent the same purification and concentration process as co-culture populations.

### Phage phenotypic assays

#### Growth rates

The thermal phenotypes of purified evolved phage populations relative to the ancestor were assessed by measuring phage and bacterial growth across an 8 h window under static incubation at 37°C and 42°C. Phage stocks were diluted to 10^5^ PFU/ml, and 300 μl was used to inoculate 2.7 ml of 10^8^ CFU/ml wild-type PAO1, with a resulting MOI = ~0.0001. This low MOI was chosen to extend phage growth curves to capture differences in phage growth rates as, at higher phage densities, both phages tend to reach carrying capacity within 2–3 h [18]. For φLUZ19, samples were taken for phage quantification at 2 h, 4 h, and 8 h. For φ14-1, samples were taken at 4 h and 8 h because preliminary assays indicated that phage growth was minimal at the 2 h timepoint. Phage quantification was performed by adding 200 μl samples to 96-well filter plates (Agilent) followed by centrifugation at 2230× *g* for 5 min before spotting onto φ14-1 or φLUZ19 resistant PAO1 double-layer overlay plates. Each fitness assay included a single replicate of each evolved phage population and three replicates of the phage ancestor. Growth rate assays were repeated three times across a 2-week period to produce three technical replicates.

#### Competitive ability

We assessed the competitiveness of evolved φ14-1 37°C and 42°C co-culture populations across time. Competitive ability was determined by growing phages under the same conditions as the fitness assay (37°C and 42°C) either alone or in the presence of an ancestral φLUZ19 competitor. For the monoculture treatment, 300 μl of phage lysate was added to 2.7 ml 10^8^ CFU/ml wild-type PAO1 stock. For the co-culture treatment, 150 μl of evolved phage stock and 150 μl of ancestral phage competitor were added. A 1:1 phage ratio was used to replicate the experimental evolution selective environment. The competition assay was conducted with two phage starting densities, 10^5^ PFU/ml (MOI = ~0.0001) and 5 × 10^8^ PFU/ml (MOI = ~5).

Phages were grown for 8 h after which samples were taken for phage quantification as previously described. Evolved φ14-1 competitiveness was determined by calculating ancestral φLUZ19 competitor growth in co-culture with evolved and ancestral φ14-1 populations against phage growth in monoculture [45]. Competition assays were repeated three times across a four-week period to produce three technical replicates.

### Phage population genomics

#### DNA extraction and sequencing

Phage DNA was extracted using a customized protocol. We used 500 μl aliquots of post-purification evolved and ancestral phage lysates (~10^10^ PFU/ml). Firstly, we added DNase (5 μl of 1000 U/ml, 5 U) to remove bacterial DNA and RNase (2 μl of 100 mg/ml, 0.2 mg) to remove RNA. Lysates were then incubated at 37°C in a heat block for 1 h and inverted every 15 min. After incubation, 67.5 μl lysis (AL) buffer and 4 μl proteinase K were added to each tube before incubating at 56°C for 15 min. After 15 min, the tubes were then incubated at 95°C for 10 min to denature the proteinase K. After denaturing, the tubes were placed on ice, and 150 μl of precipitation (N4) buffer was added. Tubes were immediately centrifuged at 13 000× *g* for 10 min to pellet cell debris, and the supernatant was transferred to a clean 2 ml Eppendorf. Cold 100% isopropanol (1.5× tube volume, ~1.1 ml) was then added, and the tubes were placed in an orbital rotator set to 10 rpm for 5 min to precipitate DNA. The tubes were centrifuged at 13 000× *g* for 20 min to pellet DNA after which the supernatant was discarded. The DNA pellet was then washed with 1 ml 70% ethanol and mixed for a further 5 min on the orbital rotator before being centrifuged again at 13 000× *g* for 20 min. This wash step was repeated twice, and after the second centrifugation step, the supernatant was discarded and the pellet dried in a heat block set to 37°C to evaporate any remaining ethanol. Finally, 30 μl of nuclease-free water was added to resuspend the pellet.

DNA purity and contamination were measured using NanoDrop 2000c (Thermo Scientific). The presence of phage DNA was confirmed using gel electrophoresis using a 100 kb ladder and a phage lambda DNA control with bands observed at the expected phage genome size. DNA was quantified using Qubit 4 (Thermofisher). Samples were diluted to DNA concentration of 50 ng/μl and sent for paired-end 2 × 250 bp read Illumina short-read sequencing with AZENTA/GENEWIZ using their Microbe-EZ pipeline. One extraction from each evolved population was sent for sequencing in addition to three extractions of each phage ancestor. Ancestral extractions were performed on single ancestral phage stocks that were used to seed all replicate populations in the evolution experiment. For ancestral and phage-resistant PAO1 bacterial genome sequencing, bacterial samples were sent to MicrobesNG for DNA extraction and hybrid (long- and short-read) sequencing.

#### Sequence analysis

Phage sequence reads were preprocessed through read trimming using Trim Galore (v.0.5.0) (https://github.com/FelixKrueger/TrimGalore) with a fastqc step and 33 phred-score read cut off. Due to high and uneven read depth, reads were downsampled using bbnorm from the bbmap package (v.39.18) (https://sourceforge.net/projects/bbmap/) to a target read depth of 1500× and a minimum depth of 1000×. Ancestral phage genomes were assembled using shovill (v1.1.0) (https://github.com/tseemann/shovill) with default parameters, and downsampled phage reads were mapped to the assemblies using Bowtie2 (v.2.3.4.2) [46] with default parameters. Read depth was checked for evenness using SAMtools (v.0.1.2) [47] view, sort, and depth functions. Ancestral phage assemblies were annotated using prokka (v.1.14.5) [48], guided by the NCBI GenBank file for each phage (φ14-1: NC_011703; φLUZ19: NC_010326). Genetic variants in phage populations were detected using breseq (v.0.36.1) [49] in polymorphism mode, using the annotated ancestral genomes as a reference. A standard polymorphism *E*-value was used as a threshold for variant calling (*E* = < 10^−2^). Only variants with >10% allele frequency (~100–150 read support) were analysed. The annotated ancestral genome was used as a reference.

Wild-type PAO1 reads were processed using an in-house pipeline. We first quality-controlled the long-reads using Filtlong (v. 0.2.1) (https://github.com/rrwick/Filtlong) with parameters --min_length 1000 --keep_percent 95. We then used Autocycler (v. 0.4.0) [50] to recover a consensus genome assembly, calling the assemblers Canu (v. 2.3) [51], Flye (v. 2.95), Miniasm (v. 0.3) [52], plassembler (v. 1.7.0) [53], and Raven (v. 1.8.3) [54]. Next, we quality-controlled the short reads using fastp (v. 0.24.2) [55], indexed with assembly with BWA (v. 0.7.19) [56], and polished with Polypolish (v. 0.6.0) [57]. Lastly, we reoriented the assembly with Dnaapler (v. 1.2.0) [58]. The workflow was deployed using a Dockerised Nextflow pipeline (v. 1.0.2) available at 10.5281/zenodo.15706447. The wild-type PAO1 assembly was annotated using prokka (v.1.14.5) [48]. φLUZ19 and φ14-1-resistant PAO1 mutations were detected by mapping long reads to the wild-type assembly with minimap2 (v.2.24) [59] and variant calling with medaka (v.2.1) (https://github.com/nanoporetech/medaka). SNPs were filtered so only those with quality scores ≥ 10 were kept.

### Statistics and data visualization

All statistical analyses and data visualization were conducted using packages in R (v.4.3.2) and RStudio [60, 61]. Data wrangling was performed using “Tidyverse” (v.2.0.0) R packages [62]. Phage growth and evolution rates were compared between evolution treatments using linear mixed effect models with the “lme4” (v.1.1-36) R package [63] where the response variable was phage density (pfu/ml) after 2 h (φLUZ19) or 4 h (φ14-1) of growth, or genetic distance from ancestor. Phage densities underwent a log_10_ transformation prior to analysis to meet statistical assumptions. Explanatory variables were an interaction term between evolution treatment and temperature, with batch as a random effect. One exception was low MOI φ14-1 growth in the presence of the φLUZ19 ancestor (Fig. S3B) where a Tweedie GLMM was used with the “glmmTMB” (v.1.1.13) R package [64] to meet model assumptions. Genetic divergence between evolved populations was calculated using principle coordinate analysis (PCoA) PERMANOVA with 10 000 permutations using the “vegan” (v.2.7.2) R package [65]. Congruence between Euclidean genetic and phenotypic distance neighbour-joining trees was calculated using Procrustes Approach to Cophylogenetic Analysis (PACo) (v.0.4.2) R package with 10 000 permutations [66].

## Results

### Rapid adaptation facilitates phage persistence in monoculture

**Figure 2:**
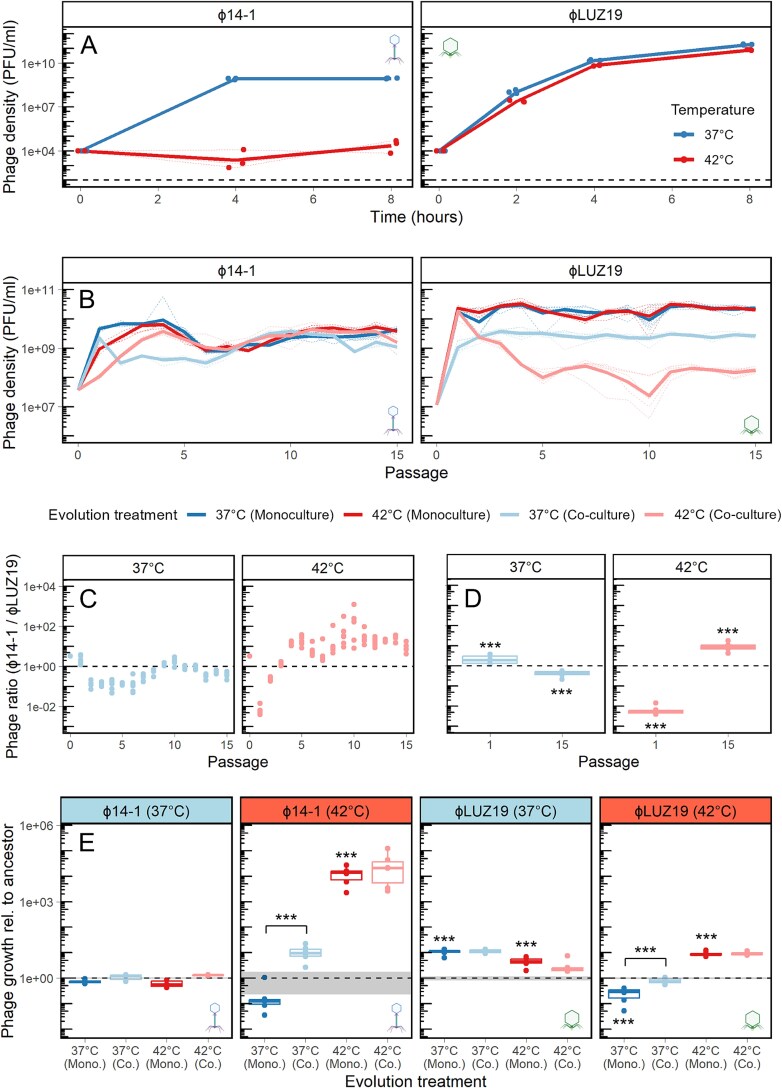
Thermal adaptation alters the competitive hierarchy. (A) Growth curves of ancestral phages across temperatures at a starting density of 10^4^ PFU/ml. Lines show ancestral phage growth curves at 37°C and 42°C. The dashed line marks the limit of detection. (B) Phage population densities under each evolution treatment across passages. Lines show phage population densities during the first 15 evolutionary passages of phages in monoculture and in co-culture. Solid lines show average values of six biological replicates (each shown as a dashed line). Values show densities at the end of each passage prior to dilution. (C) Ratio of φ14-1 to φLUZ19 densities in co-culture treatments across passages. Values show phage ratios at the end of each passage where each dot shows a replicate population. Phage ratio of 1:1 is shown with a dashed line. (D) φ14-1 to φLUZ19 ratios in co-culture treatments at passage 1 and passage 15. Asterisks show significant differences to a phage ratio of 1:1 (dashed line) where ^***^ = *P* < .001. (E) Growth rates of end-point evolved phage populations relative to the ancestral population tested in monoculture at 37°C and 42°C. φ14-1 populations were compared after 4 h growth and φLUZ19 populations were compared after 2 h growth. Evolved phage growth (box) is shown relative to ancestor (dotted line). Shaded region shows ancestor standard errors from three biological replicates, each being an average of three technical replicates. Asterisks above line connectors show significant differences between evolved populations. Asterisks above boxes show significant differences from the ancestor. ^***^ = *P* < .001. Co-culture populations were not compared to the ancestor. Otherwise, no asterisk reflects nonsignificance.

Although the ancestral φLUZ19 grows equally at 37°C and 42°C, ancestral φ14-1 populations show no signs of growth at 42°C at a starting density of 10^4^ PFU/ml (Fig. 2A). Passaging at this density would have thus resulted in dilution to extinction. We hypothesized that the thermally sensitive phage φ14-1 would avoid extinction at higher starting densities (10^7^ PFU/ml) by rapidly adapting to tolerate thermal stress through the selection of rare adaptive mutations (mutation frequency < 1/10^4^). Although φ14-1 growth was initially restricted at 42°C, it rapidly reached and maintained high densities (>10^9^ PFU/ml) across temperatures (Fig. 2B). High densities were also reached by the thermal generalist φLUZ19. Across both phages, no replicate populations went extinct.

We measured bacterial growth using optical densities at 37°C and 42°C (Fig. S4) to account for any host-mediated variation in phage densities. Without phage, *P. aeruginosa* had significantly higher growth rates at 42°C compared to 37°C (*F*_3,62_ = 78.9, *P* < .001). Based on a standard curve of optical density to colony-forming units (Fig. S1), average bacterial densities at 42°C were found to be approximately double those at 37°C across an 8 h passage. Yet, phage densities at each passage were the same or lower at 42°C than 37°C indicating that variation in phage densities could not be explained by differences in bacterial growth rates.

To determine whether φ14-1 densities were maintained at 42°C through evolutionary change, we conducted population growth assays for the end-point evolved phage populations at 37°C and 42°C (Fig. 2E). Phage population growth rates depended on an interaction between evolution treatment (37°C or 42°C) and assay temperature (φ14-1: *F*_2, 19.1_ = 199, *P* < .0001; φLUZ19: *F*_2,19.1_ = 100.0, *P* < .0001). φ14-1 populations evolved at 42°C showed a significant increase in growth at 42°C compared to ancestor (*t*(20.9) = −18.1, *P* < .0001). However, 37°C evolved populations showed no change in growth at 37°C (*t*(20.9) = 0.64, *P* = .987), likely due to phages reaching close to carrying capacity at the point of measurement (Fig. S5). φLUZ19 populations also had significantly higher growth rates at their evolved temperatures compared to the ancestor (37°C: *t*(20.9) = −7.68, *P* < .0001; 42°C: *t*(20.9) = −7.04, *P* < .0001).

Higher growth rates under evolved conditions may reflect merely adaptation to the host rather than temperature-specific fitness changes. We assessed phage growth at mismatched temperatures (Fig. 2E). φ14-1 evolved populations showed no improvement in growth at mismatched temperatures (37°C evolved populations: *t*(20.9) = 2.37, *P* = .212, 42°C evolved populations: *t*(20.9) = 1.00, *P* = .912). In addition, φLUZ19 populations evolved at 37°C exhibited a significant growth rate decrease at 42°C (*t*(20.9) = 5.13, *P* < .001). However, φLUZ19 populations evolved at 42°C were found to also have significantly higher growth rates at 37°C than ancestor (*t*(20.9) = −4.79, *P* < .01) indicative of adaptation to the host as opposed to the thermal regime in this instance.

### Thermal adaptation alters the competitive hierarchy

Even though thermal adaptation occurred in monoculture, we hypothesized that adaptation would be restricted in co-cultures due to evolutionary constraint from interphage competition [5, 31]. Both φ14-1 and φLUZ19 were initially found to have lower population densities in co-culture than in monoculture (Fig. 2B). At 37°C, φ14-1 and φLUZ19 had ~10-fold lower densities in co-culture up to passage 6, after which φLUZ19 densities remained suppressed and φ14-1 densities converged with those of the monoculture populations. Even though φ14-1 was heavily restricted by φLUZ19 in early passages at 42°C, it reached similar densities to monoculture populations by passage 4. In contrast, φLUZ19 co-culture densities were initially high at 42°C but rapidly decreased before stabilizing.

We hypothesized that the population decline in φLUZ19 42°C co-culture populations occurred due to a shift in the competitive equilibrium with φ14-1 following thermal adaptation. We assessed the competition dynamics by tracking the ratio of φ14-1 and φLUZ19 densities across co-culture passages where a ratio > 1 reflects a φ14-1 competitive advantage and vice versa. At 42°C, the ratio of φ14-1 densities relative to φLUZ19 fell in the initial passage but then rapidly increased before stabilizing at passage 5 (Fig. 2C). The phage ratio conversely remained relatively stable across all passages in the control. By analysing phage ratios at passages 1 and 15, we found that, at 42°C, φ14-1 had a significant competitive disadvantage at passage 1 (*t*(19.9) = −27.5, *P* < .001) but a significant advantage by passage 15 (*t*(19.9) = 11.7, *P* < .001). φ14-1 competitive advantage showed a small decrease between passage 1 and 15 in the control (passage 1: *t*(19.9) = 3.9, *P* < .001; passage 15: *t*(19.9) = −4.7, *P* < .001).

We further investigated phage competitive profiles in time-shifted, direct competition assays between ancestral and evolved co-culture populations against an ancestral competitor population (Fig. S3). These assays were repeated at low and high MOIs due to previous studies indicating that phage competitive fitness may be MOI-dependent [67, 68]. At low MOI, the restriction of φLUZ19 ancestral growth by φ14-1 at 42°C was significantly weaker in the φ14-1 37°C co-culture population compared to the φ14-1 ancestor or 42°C co-culture population (ancestor: *t*(21.6) = −4.4, *P* < .001; 42°C co-culture: *t*(19.2) = −6.3, *P* < .001) (Fig. S3A). In contrast, at high MOI, restriction of φLUZ19 at 42°C was significantly greater in the φ14-1 37°C and 42°C co-culture populations compared to the φ14-1 ancestor (37°C co-culture: *t*(21.6) = 2.9, *P* < .05; 42°C co-culture: *t*(21.6) = 4.0, *P* < .01), although the magnitude of change was relatively small. The restriction of φ14-1 ancestral growth by φLUZ19 at 37°C was significantly greater in the φLUZ19 37°C co-culture population compared to ancestor at low MOI (GLMM: *Z* = 4.7, *P* < .001) and greater than the 42°C co-culture population at both low and high MOIs (GLMM, low MOI: *Z* = −3.4, *P* < .01; high MOI: *t*(19.1) = −2.8, *P* < .05) (Fig. S3B). There was no significant difference in ancestral competitor restriction between φLUZ19 populations at 37°C or φ14-1 populations at 42°C. The φ14-1 competitive advantage in 42°C co-culture populations did not reflect an escalating increase in competitive fitness across evolutionary time, or a progressive loss in competitive fitness by the φLUZ19 42°C co-culture competitor.

By restricting phage growth, we hypothesized that the presence of a competitor would constrain phage thermal adaptation. We assessed thermal adaptation by comparing phage thermal phenotypes in monoculture and co-culture evolved populations (Fig. 2E). For both phages, we found that growth rates depended on an interaction between evolution treatment (monoculture and co-culture) and assay temperature (φ14-1: *F*_4,39_ = 132, *P* < .0001; φLUZ19: *F*_4,39_ = 108, *P* < .0001). There was no significant difference in growth rates between monoculture and co-culture evolved populations at their evolved temperatures (37°C - φ14-1: *t*(39) = −0.87, *P* = .99; φLUZ19: *t*(39) = 0.38, *P* = 1.0; 42°C - φ14-1: *t*(39) = −1.1, *P* = .98; φLUZ19: *t*(39) = −0.19, *P* = 1.0). Phages evolved at 37°C in co-culture showed significantly higher fitness at 42°C than those evolved in monoculture (φ14-1: *t*(39) = −9.19, *P* < .0001; φLUZ19: *t*(39) = −6.04, *P* < .0001).

### Temperature and competition select for mutations in tail proteins and replication machinery

**Figure 3:**
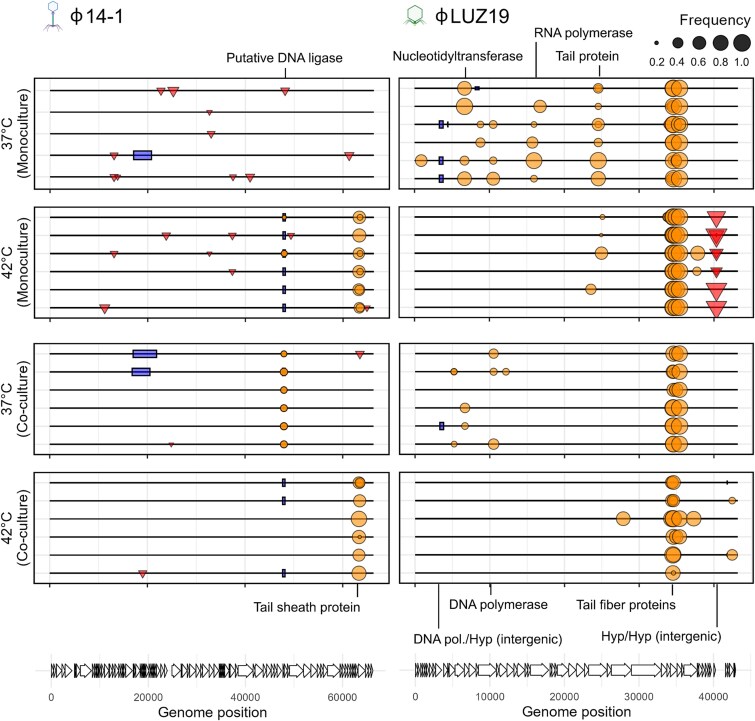
Competition and temperature select for mutations in tail proteins and replication machinery. Mutation plots show genetic variants associated with thermal adaptation and competition in phage populations. Lines represent individual biological replicates. Symbols within plots show variants across the phage genome at >20% prevalence, which were not observed in the ancestral population. Symbols reflect mutation type where circle = SNP, box = deletion, inverted triangle = insertion. Length of deletion bars represents the size of deletion except for the φ14-1 deletion at ~48 kb, which is a 1 bp deletion but given a fixed size for visibility. Labels show annotations for genes that contain mutations in at least three replicate populations in the same evolution treatment, reflecting parallel evolution. All putative adaptive variants are presented in Table S2. Putative DNA ligase in φ14-1 was originally annotated as a hypothetical protein but has high homology to Pseudomonas phage PhL_UNISO_PA-DSM_ph0031 DNA ligase protein.

To further understand the genomic causes of adaptation to temperature and competition, we conducted phage population sequencing and determined the identity and frequency of genetic variants (Fig. 3; Table S1). Putative adaptive variants were defined as those with a frequency > 20% and which occurred in genes that acquired mutations in at least two biological replicates across all treatments (Table S2). No mutations found with frequency > 20% in the ancestral population were found to have increased in frequency in the evolved populations; detected mutations were either rare in the ancestral population or were acquired de novo during the selection experiment.

As φLUZ19 growth is restricted by slow host attachment rates [18], we hypothesized that φLUZ19 mutations would occur in tail proteins responsible for attachment. The most prominent genetic changes in the φLUZ19 evolved populations were a series of high-frequency SNPs in two genes encoding a tail protein and tail fibre protein. Although the tail protein mutations were only found in monoculture populations, tail fibre protein mutations were found across all temperature and phage combination treatments, suggesting that these reflect adaptation to the host rather than to interspecific competition or thermal stress. φLUZ19 42°C monoculture evolved populations also all contained insertions in an intergenic region between two hypothetical proteins, suggesting that altered regulation of gene expression may also contribute to thermal adaptation in φLUZ19. These insertions were not observed in co-culture populations, indicating competition may have restricted the acquisition of adaptive mutations. Other mutations were exclusively found in 37°C monoculture evolved populations and included putative bacterial immune system-associated nucleotidyltransferase [69], RNA polymerase, and DNA polymerase mutations. These mutations may contribute to φLUZ19 low temperature adaptation by increasing phage replication within cells.

φ14-1 adaptation at 42°C was linked to a series of high frequency SNPs in a gene encoding the phage tail sheath, a phage component involved in DNA transfer into bacterial cells [70]. We also observed parallel deletions and SNPs in the 42°C monoculture and 37°C co-culture populations in a putative DNA ligase (BlastP: 97.47% identity, 95% sequence overlap with Pseudomonas phage PhL_UNISO_PA-DSM_ph0031 DNA ligase protein), a protein that is essential for phage DNA replication and fitness [71, 72]. These results imply that competition and high temperature co-select for altered DNA replication in φ14-1.

### Thermal stress and competition shape phage molecular evolution

**Figure 4:**
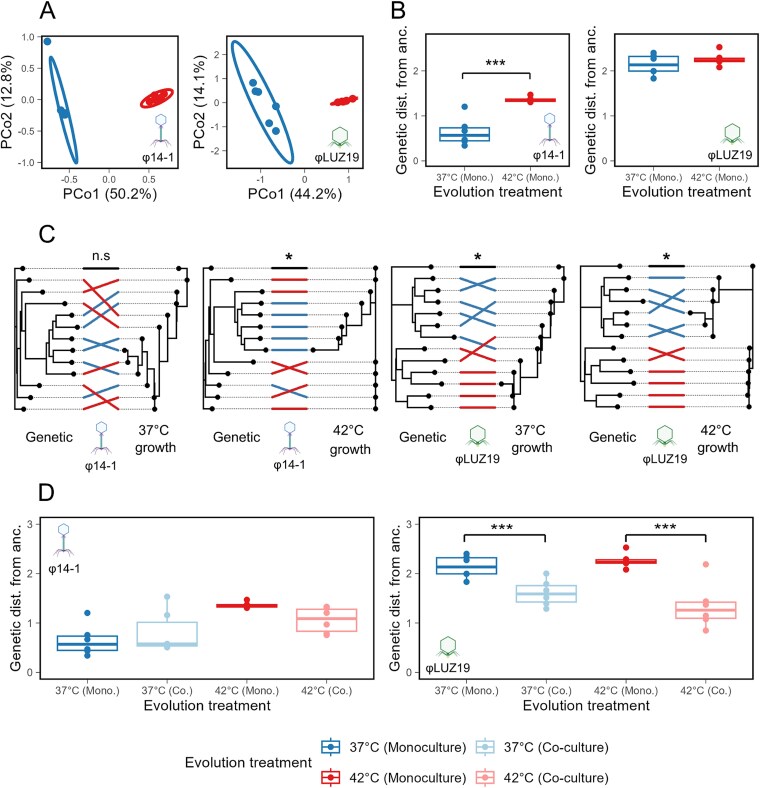
Thermal stress accelerates and competition constrains phage molecular evolution. (A) Genomic divergence between evolved monoculture phage populations. PCoA plots show Euclidean genetic distance clustering between 37°C and 42°C evolved phage populations based on mutation position and frequency. (B) Evolution rates of evolved monoculture phage populations based on the Euclidean genetic distance from ancestor. ^***^ = *P* < .001. No asterisk reflects nonsignificance. (C) Congruence analysis of neighbour-joining trees constructed based on Euclidean genetic distances (left-hand plots, labelled “genetic”) and Euclidean distances based on phage growth rates at 37°C and 42°C (right-hand plots, labelled “37°C growth” and “42°C growth,” respectively). Congruence is shown by the alignment of tips corresponding to individual replicate populations between trees. High congruence is shown by few cross-overs. Trees are rooted using the ancestral phage. (D) Evolution rates of evolved monoculture phage populations compared to evolved co-culture populations. Evolution rates are determined based on Euclidean genetic distance from ancestor. ^***^ = *P* < .001. No asterisk reflects nonsignificance.

Phage populations evolved at high temperatures differed from control populations both in terms of thermal phenotypes and the mutations they acquired. We hypothesized that 37°C and 42°C evolved populations would also diverge at the whole-genome level due to differing evolutionary trajectories. PCoA analysis based on phage Euclidean genetic distances showed that both φ14-1 and φLUZ19 had significant genetic divergence between 37°C and 42°C evolved populations (φ14-1 PERMANOVA: *F* = 9.9, *R*^2^ = 0.50, *P* < .01; φLUZ19: PERMANOVA: *F* = 7.6, *R*^2^ = 0.43, *P* < .01) (Fig. 4A). We then hypothesized that genetic distance from ancestor would be highest in the populations that experienced the greatest change in growth rates. φ14-1 had significantly higher genetic distances at 42°C than at 37°C (*F*_1,10_ = 30.3, *P* < .001) (Fig. 4B). No significant difference in genetic distance was observed between temperatures for φLUZ19 populations (*F*_1,10_ = 1.2, *P* < .30).

As phages showed both phenotypic and genomic divergence based on thermal regime, we hypothesized that genetically similar phage populations may have similar thermal fitness. We assessed the relationship between thermal fitness and genomic change by measuring congruence between neighbour-joining trees constructed based on Euclidean phenotypic and genetic distances (Fig. 4C). Highly significant congruence was observed for both phages based on growth rates at 42°C (φ14-1: M^2^_xy_ = 5.8, *P* < .01; φLUZ19: M^2^_xy_ = 15.9, *P* < .001). For 37°C growth, significant congruence was observed for φLUZ19 (M^2^_xy_ = 20.4, *P* < .05) but not for φ14-1 (M^2^_xy_ = 8.1, *P* = .18).

Given the evolutionary constraint of competition, we also hypothesized that co-culture evolved populations would diverge from monoculture evolved populations and have lower genetic distances from ancestor. Significant genomic divergence was observed between monoculture and co-culture populations for φ14-1 at 37°C and for φLUZ19 at 42°C (φ14-1: PERMANOVA: *F* = 2.3, *R*^2^ = 0.19, *P* < .01; φLUZ19: PERMANOVA: *F* = 6.9, *R*^2^ = 0.4, *P* < .01). Divergence was not significant for φ14-1 at 42°C or for φLUZ19 at 37°C (φ14-1: PERMANOVA: *F* = 2.3, *R*^2^ = 0.19, *P* = .07; φLUZ19: PERMANOVA: *F* = 1.7, *R*^2^ = 0.15, *P* = .07) (Fig. S6). φ14-1 populations had similar genetic distances in monoculture and co-culture (37°C: *t*(20) = −0.972, *P* = .77; 42°C: *t*(20) = 1.77, *P* = .31). However, φLUZ19 evolved populations had significantly lower genetic distances in co-culture compared to monoculture at both 37°C and 42°C (37°C: *t*(20) = 3.1, *P* < .05; 42°C: *t*(20) = 5.3, *P* < .001) (Fig. 4D).

## Discussion

Environmental stress alters communities by restricting the growth of sensitive community members and destabilizing competitive hierarchies [29]. We show that the thermally sensitive φ14-1 phage can rapidly adapt to thermal stress, even in the presence of a thermally tolerant φLUZ19 phage competitor. We further found that competition at permissive temperatures, where both phages grow efficiently, can drive the evolution of elevated φ14-1 thermal tolerance. These findings support previous studies by demonstrating that phages, at least with nonevolving hosts [73], are highly evolvable in response to environmental stress [17, 27, 28]. The ability of φ14-1 to adapt to thermal stress at high but not low densities is consistent with evolutionary rescue. However, poor φ14-1 growth at low densities may alternatively reflect ecological processes such as Allee effects [74] or MOI-dependent infection success [75]. The results nevertheless contradict findings that competitive interactions constrain environmental adaptation by reducing growth rates and mutational supply [5, 31]. One potential explanation is that, although strong competition may constrain evolution, weak or moderate competition may have increased the strength of selection for φ14-1 thermal adaptation [4, 29, 76]. Alternatively, adaptation may have been driven through co-selection by competition and temperature for the same traits. We identified mutations in the same genes in φ14-1 populations evolving under both high temperature selection and at permissive temperatures in the presence of competition. The evolution of stress tolerance in free-living organisms has historically been associated with trade-offs in competitive fitness [77–79]. Increased selection or beneficial pleiotropy could mean that competition in phage systems leads to trade-ups rather than trade-offs with adaptation to environmental stress.

Evolutionary rescue has largely been thought to restrict biodiversity loss by preventing taxa from becoming extinct [8]. Within communities, however, we found that rapid thermal adaptation can cause one species to become a superior competitor, thereby promoting the competitive suppression of others. As competitor population size declines, the risk of population collapse increases [80]. Rapid adaptation, and so evolutionary rescue, may thus be insufficient to prevent biodiversity loss under environmental stress. Adaptation to environmental stress can come at an ecological cost where increased environmental tolerance leads to a trade-off with growth rates [81, 82]. However, ecological destabilization can also occur via trade-ups if growth rates increase in the recently adapted population [12, 13]. We showed that even though the rescued phage at 42°C gained a competitive advantage over its sympatric competitor, rescued phage competitiveness did not escalate across evolutionary time and was not elevated against the ancestral competitor. Given the co-culture φLUZ19 populations did not show a progressive loss of competitiveness against the ancestral competitor, φ14-1 competitive dominance may be specific to co-evolving competitors. By reducing competitor population densities, rapid thermal adaptation had the additional impact of constraining competitor evolution rates and restricting the acquisition of putative adaptive mutations. Environmental adaptation may therefore cause community instability by both depressing competitor population densities and limiting community adaptability in response to future environmental stress [5].

Despite being suppressed by the newly dominant φ14-1, φLUZ19 competitor phage populations ultimately stabilized at reduced densities. Modern co-existence theory states that co-existence can occur through stabilizing mechanisms such as niche differences or through competitors having similar relative fitness [22]. Phage co-existence has previously been attributed to variation in host cell susceptibility to infection [23]. In our system, φ14-1 and φLUZ19 use different bacterial surface receptors to infect cells [35, 83]. We propose that heterogeneity in the expression of phage receptors in the bacterial population [84] may have created a niche that enabled φLUZ19 persistence, but which was inaccessible to the rescued φ14-1 population. Alternatively, phage fitness differences may have been resolved through φLUZ19 co-evolution. For example, φLUZ19 acquired tail fibre and tail protein mutations, which likely contribute to host attachment rates and within-host competitiveness [85]. Even though the exact cause of co-existence remains unclear, the results highlight that stabilizing mechanisms could buffer against total competitive exclusions that arise through evolutionary rescue.

Global biodiversity is decreasing due to environmental stress caused by land use change, pollution, and climate change [16, 86, 87]. This study highlights a process by which environmental adaptation and possibly evolutionary rescue, a force typically associated with preserving biodiversity, can make the community less resilient over ecological and evolutionary time. That parasites, and specifically viruses, can rapidly adapt to environmental stress has implications for our understanding of how parasites might evolve in the context of novel and hostile environments, such as following spillover events [88] or in hosts treated for infection [89]. A loss of parasite diversity could increase the survival rates of some host species [90]; there could be a reduced burden of infection and fewer co-infections. Although within-host competition has been associated with selection for low virulence phage cheats [67, 68], there may also be weaker overall selection favouring virulence due to less interspecific competition [91]. Given parasites are beneficial for keeping pest or pathogenic hosts (as in this study) at bay [89, 92], lower diversity would have negative consequences for animal, plant, and ecosystem health. Ultimately, consideration of the eco-evolutionary dynamics will help us better understand how communities will respond to increasingly frequent environmental stressors in a changing world.

## Supplementary Material

Supplementary_material_wrag114

## Data Availability

The R code used and datasets generated during and/or analysed during the current study are available on GitHub at: https://github.com/SamuelGreenrod/Phage_thermal_adaptation. Phage sequence reads are accessible on NCBI (https://www.ncbi.nlm.nih.gov/) under BioProject ID: PRJNA1332698. Bacterial sequence reads are available on NCBI under BioProject ID: PRJNA1332799.
