# Supplementary material for: Rapid adaptation accelerates competitive suppression in a parasite community
Source: ISME J. 2026 Jun 13;20(1):wrag114. doi: 10.1093/ismejo/wrag114 (PMC13374857; doi:10.1093/ismejo/wrag114)
Supplement: Supplementary_material_wrag114 [file supplementary_material_wrag114.zip › Supplementary_figures_wrag114.docx]

**Evolutionary rescue accelerates competitive suppression in a parasite community**

**Samuel TE Greenrod^1#^, Daniel Cazares^1^, Weronika Ślesak^1^, Tobias E Hector^1^, R. Craig MacLean^1,2^, Kayla C King^1,3,4,#^**

^1^ Department of Biology, University of Oxford, Oxford, UK

^2^ All Souls College, High Street, Oxford OX1 4AL, UK

^3^ Department of Zoology, University of British Columbia, Vancouver, Canada

^4^ Department of Microbiology & Immunology, University of British Columbia, Vancouver, Canada

^#^Correspondence: [greenrodsam@gmail.com](mailto:greenrodsam@gmail.com) (Samuel Greenrod); [kayla.king@ubc.ca](mailto:kayla.king@ubc.ca) (Kayla King)

**Extended Data**


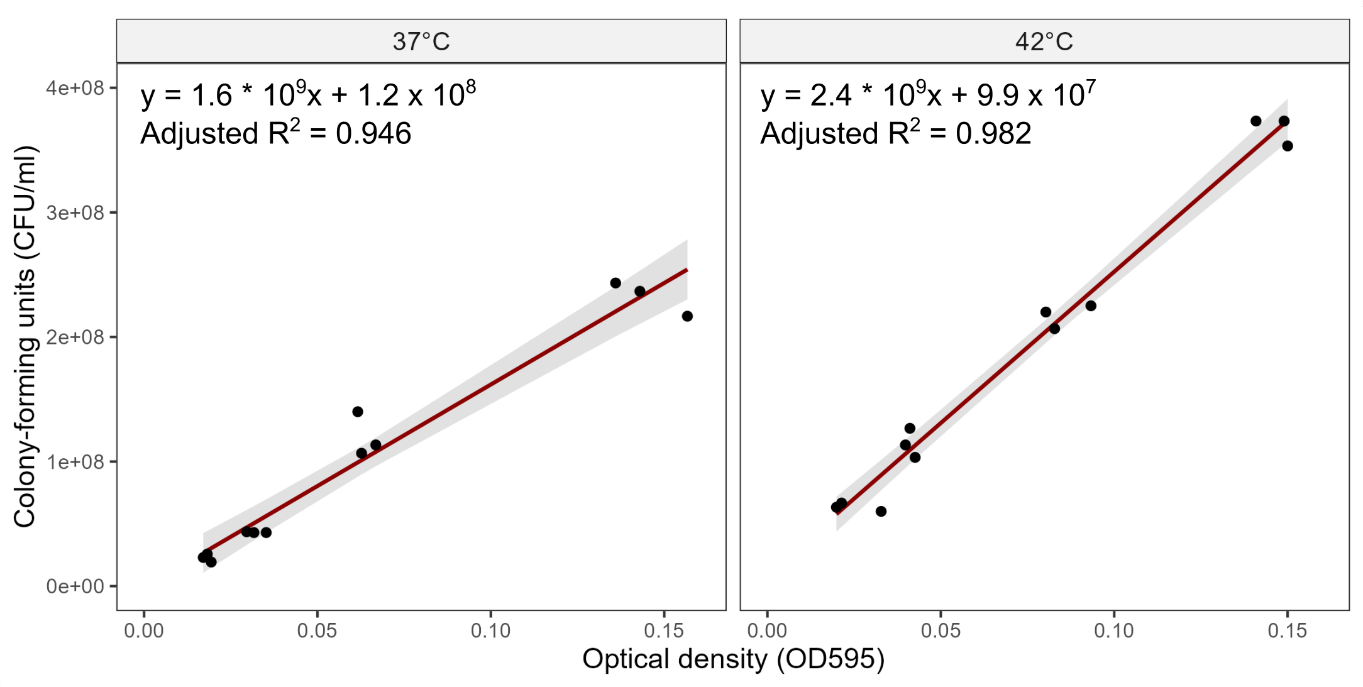


**Figure S1.** **Optical density v colony-forming units standard curve.** A regression of optical density and colony-forming units was used to determine the optical density of *P. aeruginosa* PAO1 overnights required to have ~10^8^ CFU/ml.


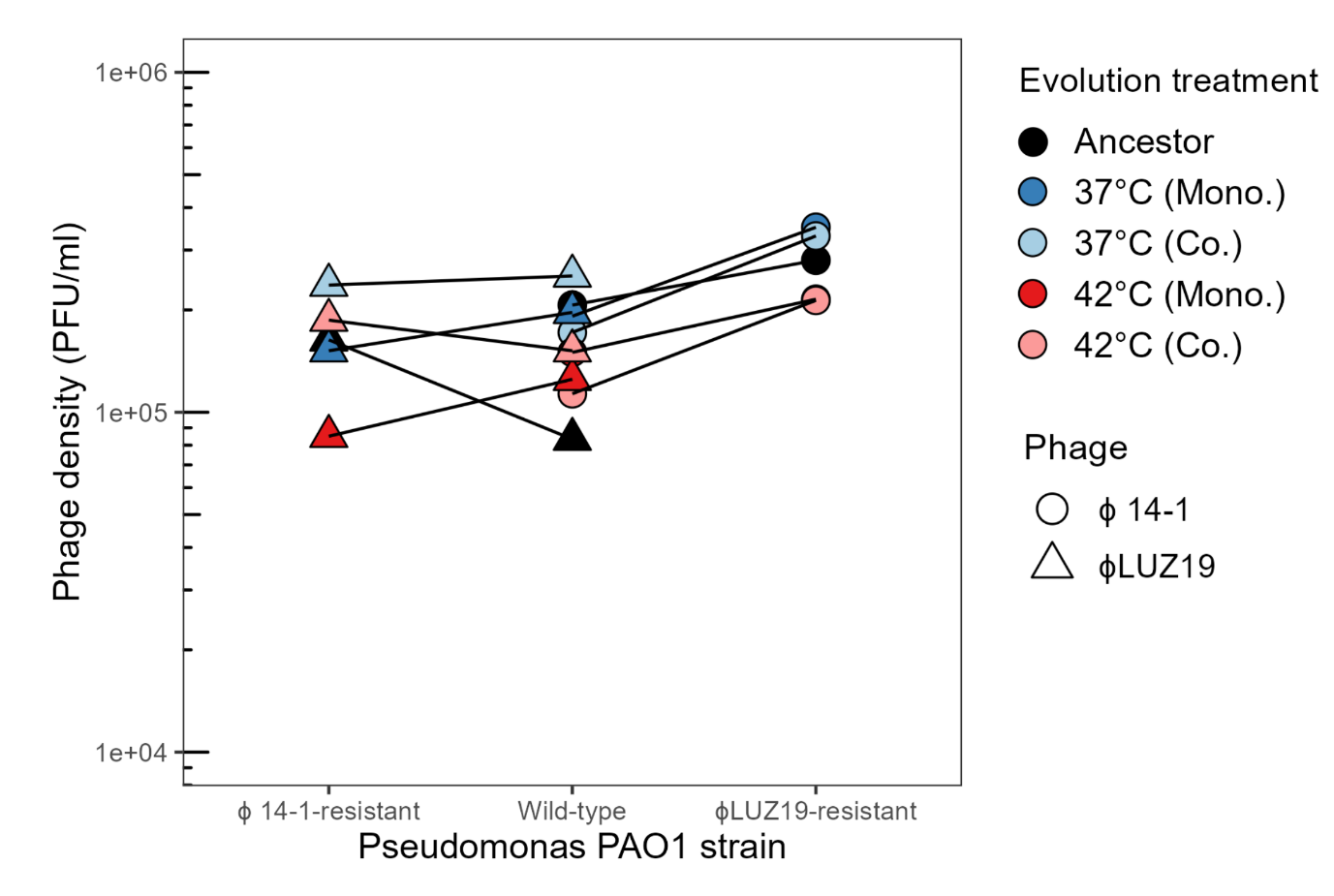


**Figure S2. Efficiency of plaque formation is the same for evolved and ancestral phage populations.** Plot shows the density of evolved and ancestral phage lysates as measured with plaque assays on the wild-type and resistant PAO1 strains. Lines between points show phage counts on the same stocks on separate hosts.


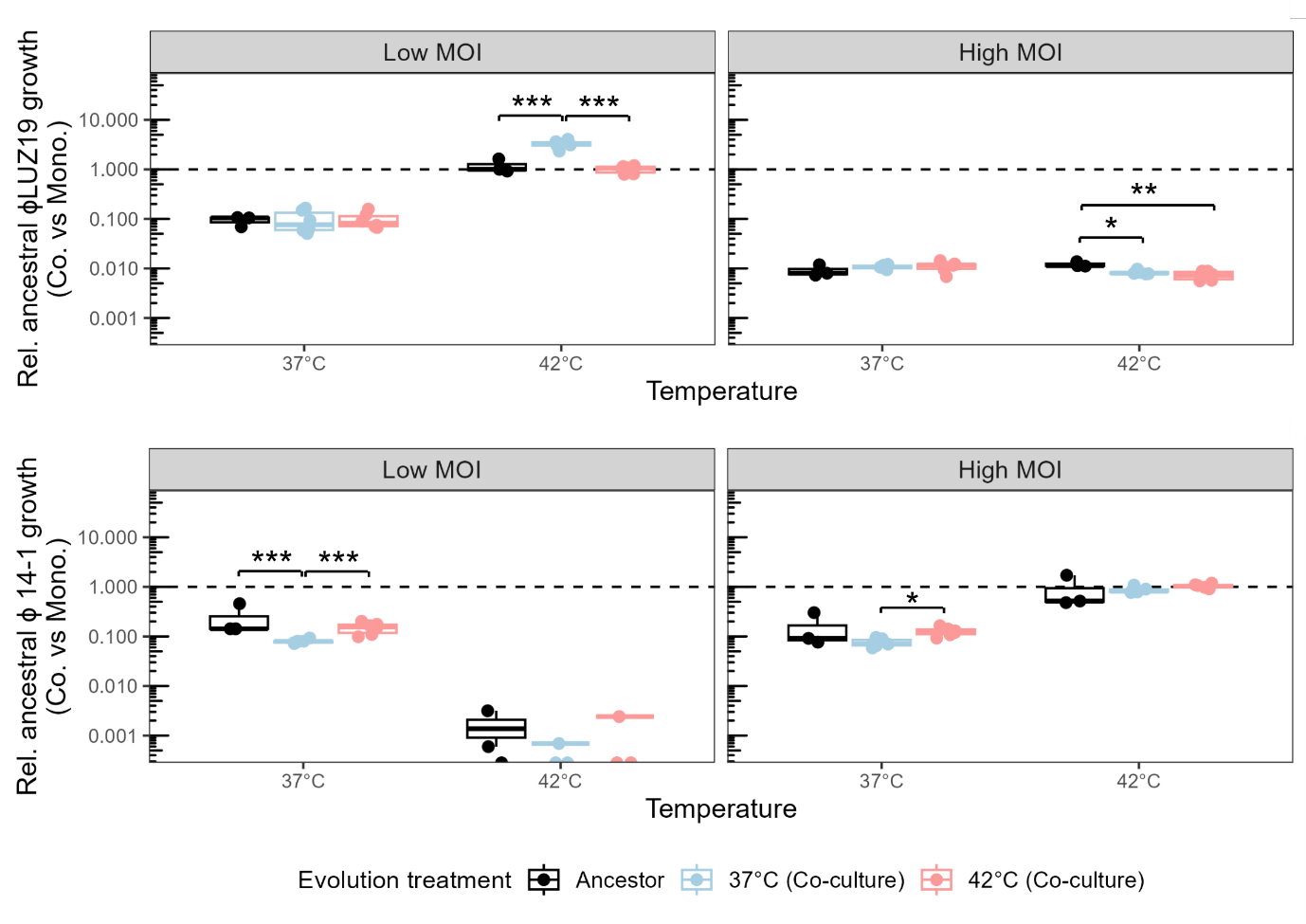


**Figure S3. Co-culture evolved populations have similar competitiveness to ancestor. A**) Boxplots show the growth of ancestral φLUZ19 at 37°C and 42°C in co-culture with the ancestral and co-culture evolved φ14-1 populations relative to growth in monoculture. **B**) Boxplots show the growth of ancestral φ14-1 at 37°C and 42°C in co-culture with the ancestral and co-culture evolved φLUZ19 populations relative to growth in monoculture. Ancestral and co-culture evolved population competitiveness was determined at both low MOI (MOI = 0.0001) and high MOI (MOI = 5). Values below 1 (dashed black line) indicate measured phage growth (φLUZ19 in **A**, φ14-1 in **B**) is inhibited by the presence of competitor populations. Asterisks show significant differences in growth restriction between evolution treatments. * = p < 0.05, ** = p < 0.01, *** = p < 0.001.


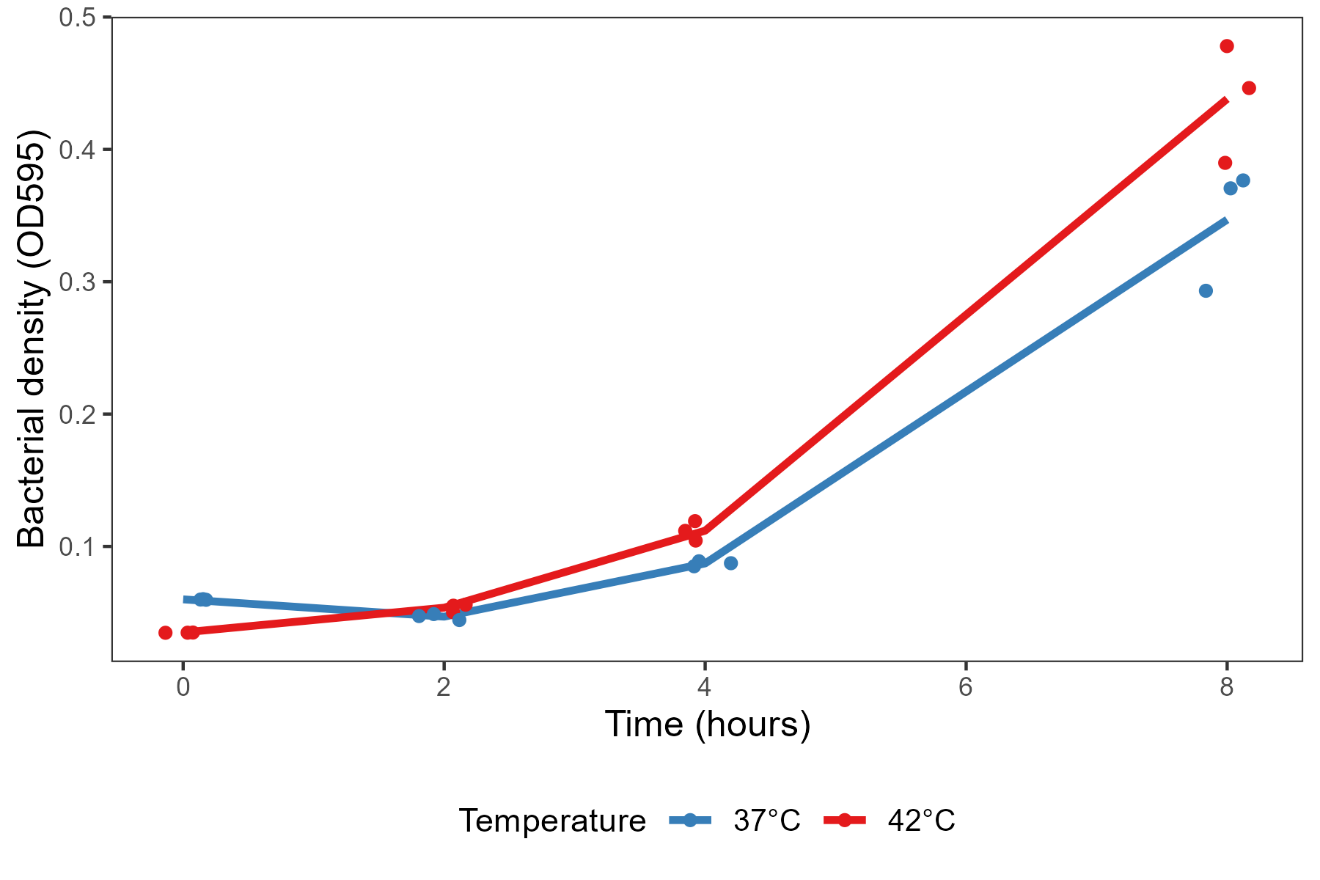


**Figure S4. Bacterial growth in absence of phage is similar across temperature.** Growth curves of no phage bacterial control. Dots reflect an average of three technical replicates. Bacterial growth was measured three separate times.


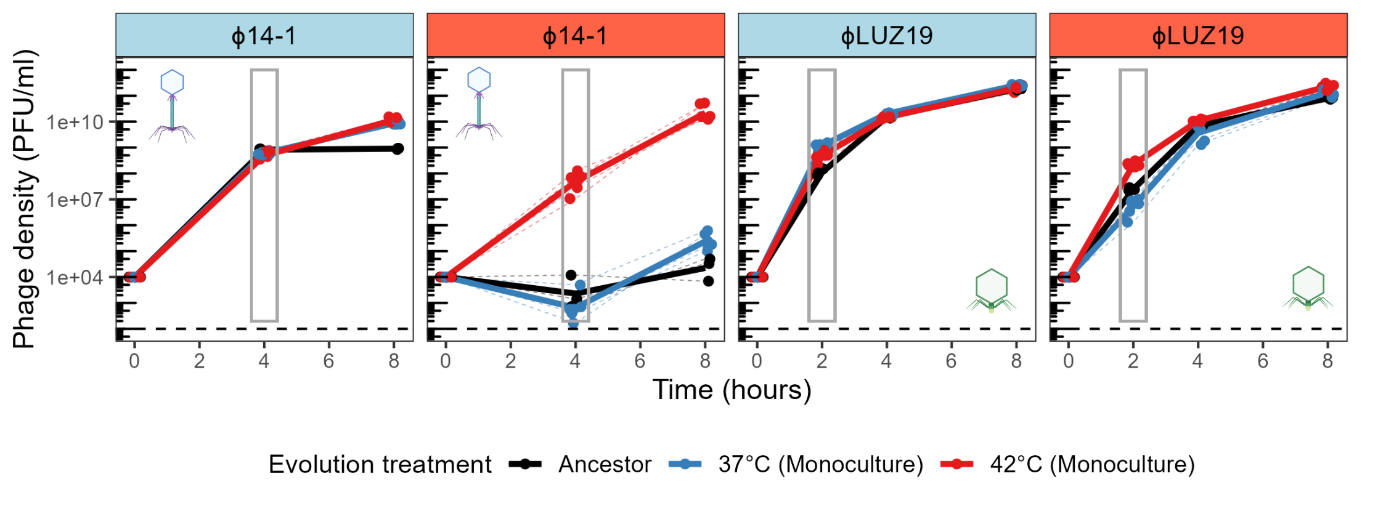


**Figure S5. Evolved and ancestral phage growth curves at 37°C and 42°C**. Grey box shows time points selected for fitness comparison. Black dashed line shows lower detection limit. Phage growth was assessed at 37°C (light blue strip) and 42°C (light red strip).

**
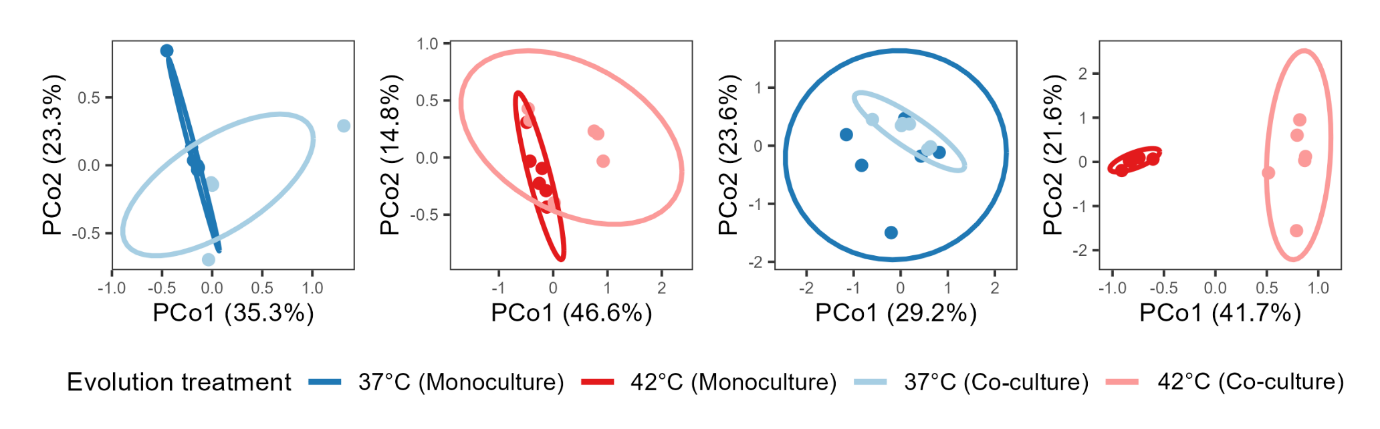
**

**Figure S6. Phages show significant genetic divergence between monoculture and co-culture populations.** PCoA plots show Euclidean genetic distance clustering between monoculture (37°C in deep blue, 42°C in deep red) and co-culture (37°C in light blue, 42°C in light red) evolved populations.
